# Adaptation of the Training Resource Package to Strengthen Preservice Family Planning Training for Nurses and Midwives in Tanzania and Uganda

**DOI:** 10.9745/GHSP-D-18-00030

**Published:** 2018-10-03

**Authors:** Stembile Mugore, Mercy Mwanja, Vumilia Mmari, Alphonce Kalula

**Affiliations:** a Evidence to Action Project, IntraHealth International, Washington, DC, USA.; b Uganda Nurses Midwives Council, Kampala, Uganda.; c Tanzania Ministry of Health, Dar es Salaam, Tanzania.; d East, Central and Southern Africa Health Community College of Nursing (ECSACON), Arusha, Tanzania.

## Abstract

**Background:**

Tanzania and Uganda have high total fertility and maternal mortality rates, and low contraceptive prevalence rates. High-quality preservice family planning education for nurses and midwives can improve the quality of sexual and reproductive health care, thereby improving health outcomes.

**Description of Intervention:**

In 2015, we worked with relevant stakeholders in Tanzania and Uganda through a series of surveys, assessments, and workshops to adapt modules of the Training Resource Package for Family Planning (TRP), an evidence-based global resource, to improve the quality of preservice family planning education for nurses and midwives. With support, a wide range of stakeholders, including policy makers, program managers, educators from nursing and midwifery training institutions, and representatives from professional associations, identified relevant TRP modules and adapted them to each country's context to inform and develop their own lesson plans in accordance with national policies, guidelines, and standardized preservice education templates.

**Lessons Learned:**

Important lessons from the adaptation process include the following: (1) engage relevant ministries of health and education, professional associations, and regulatory councils at each step of the process to increase the acceptability and utility of the TRP; (2) use a context-specific process for adaptation of the TRP, as not one process will fit the needs of all countries; and (3) include nursing and midwifery educators in the adaptation process to create an established pool of trainers who can then cascade the TRP to other educators in their respective schools. Overall, participants in both countries expressed challenges with incorporating competency-based teaching methods into their curricula because they were unfamiliar with such approaches themselves and with reducing the extensive TRP content to fit within the time constraints for preservice education.

**Conclusion:**

Adaptation of an evidence-based global family planning training resource in Tanzania and Uganda resulted in substantive changes to the curricula of the reproductive health preservice course unit that will support nurses and midwives to provide quality, rights-based family planning services.

## Abstract (precis)

Lessons learned when adapting the evidence-based global family planning training resource package included the need to: (1) engage key nursing and midwifery educators for buy-in; (2) update the technical skills of educators in contraceptive technology and competency-based training methods; and (3) adapt to the local context including condensing the global content for the time-limited preservice education context.

## BACKGROUND

The modern contraceptive prevalence rate in Tanzania and Uganda, 32% and 35%, respectively, is low relative to that in high-income countries, whereas the maternal mortality rate, 556 and 336 per 100,000 live births, respectively, is high.^1^^,^^2^ Tanzanian women have, on average, 5.2 children, and Ugandan women 5.4.^1^^,^^2^ Across both countries, approximately 1 in 4 women has an unmet need for contraception.^1^^,^^2^ Quality family planning services provided by well-trained, competent health workers can lead to increased uptake of contraception and reductions in unintended pregnancies, in turn leading to improved health outcomes.

Preservice education (i.e., training and instruction provided to health professionals in an educational setting before they begin their careers) is an important building block for equipping health workers with the skills and knowledge necessary to provide high-quality care.^3^ Preservice education is often more cost-effective than in-service training because preservice education usually trains large swathes of health workers at once.^4^ The skills and knowledge imparted during high-quality preservice education can also be more sustainable than during in-service training, as participants may be more receptive to training when they are learning the bedrock skills for their future careers.^4^

Preservice education, which usually trains large swathes of health workers at once, is often more cost-effective than in-service training.

Despite its importance, data suggest that the quality of preservice family planning education in Tanzania and Uganda is subpar. For example, a 2013 survey of 70 nursing and midwifery professionals across East, Central, and Southern Africa revealed that many of the nursing and midwifery schools in these countries have limited materials and technical expertise.^5^ As one 2014 study of 35 preservice schools in Tanzania concluded, “Pre-service FP [family planning] teaching in Tanzania is theoretical, poorly guided, and skewed toward short-acting methods; a majority of the schools are unable to produce competent FP service providers.”^6^

## DESCRIPTION OF INTERVENTION

We worked with relevant stakeholders in Tanzania and Uganda to improve the quality of preservice family planning education for nurses and midwives. Stakeholders included the East, Central, and Southern Africa Community (ECSA), an organization focused on improving health across this region, as well as the Tanzanian and Ugandan governments. We focused on nurses and midwives because they comprise the majority of the professional health workforce.^7^ In Tanzania and Uganda, approximately 200 public and private schools train the 2,400 to 3,000 nursing and midwifery students who graduate each year.^4^

We worked with relevant stakeholders in Tanzania and Uganda to improve the quality of preservice family planning education for nurses and midwives.

To enhance the quality of preservice family planning education for these cadres of health workers, we adapted the Training Resource Package for Family Planning (TRP)—a comprehensive set of family planning curricula—to meet the needs of the 2 countries, and supported use of the adapted curricula during preservice education (Figure).

**FIGURE:**
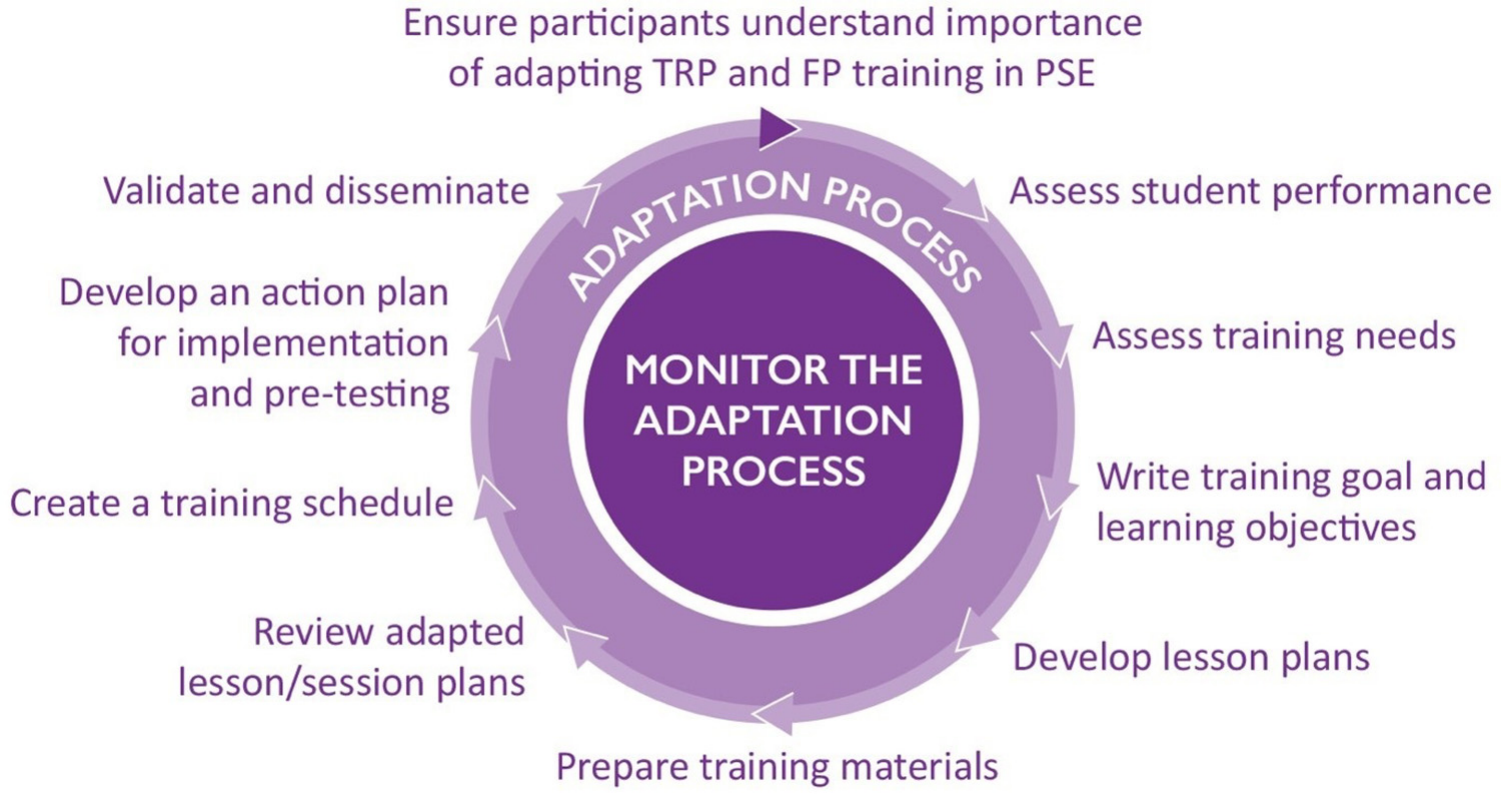
Illustrative Representation of the TRP Adaptation Process Abbreviations: FP, family planning; PSE, preservice education; TRP, Training Resource Package for Family Planning.

### The Training Resource Package for Family Planning

Developed in 2012 by the U.S. Agency for International Development (USAID), the United Nations Population Fund (UNFPA), and the World Health Organization (WHO), along with a number of sexual and reproductive health organizations, the TRP contains 13 modules, each outlining a particular topic. The topics include information on contraceptive methods, comprehensive contraceptive counseling, information on side effects, and youth-friendly contraceptive services (Box). Each module comes with a lesson plan, PowerPoint presentations, and additional resources and activities that instructors might find useful for relaying information to students. The TRP is available for free online and can be accessed at: https://www.fptraining.org/. The TRP uses the most up-to-date technical information to inform the content of its modules.

BOX **List of Modules Included in the Training Resource Package for Family Planning**

1. Benefits of Family Planning
2. Combined Oral Contraceptives
3. Condoms – Female
4. Condoms – Male
5. Contraceptive Implants
6. Emergency Contraceptive Pills
7. Emergency Contraceptive Pills Training for Pharmacists
8. Family Planning Counseling
9. Intrauterine Devices
10. Lactational Amenorrhea Method
11. Progestin-Only Injectable Contraceptives (Injectables)
12. Standard Days Method
13. WHO's Family Planning Guidance Documents and Job Aids

Our process, results, and lessons learned are further detailed in the following sections.

### Dissemination Through ECSA

In September 2014, ECSA held its 5th quadrennial general meeting in Harare, Zimbabwe, which was attended by representatives of ECSACON (ECSA's nursing- and midwifery-focused arm), development partners, nursing and midwifery educators, regulatory councils, professional associations, service delivery managers, providers, and students. The theme of this meeting was increasing access to quality nursing and midwifery care across ECSA's member states (i.e., Kenya, Lesotho, Malawi, Mauritius, Swaziland, United Republic of Tanzania, Uganda, Zambia, and Zimbabwe).

In the days preceding the meeting, we hosted a half-day workshop with educators from ECSA member states during which we disseminated the TRP, demonstrated its use for in-service training, and discussed adaptation for preservice education. Participants at this half-day preconference workshop included tutors from preservice education of nurses and midwives, representatives of regulatory councils and professional associations, students, service delivery managers from ECSA and nonmember countries Botswana, Namibia, and South Africa. We demonstrated how to access the TRP online and oriented them on how it is structured. Participants reviewed the TRP counseling module and engaged in role-play exercises to demonstrate the module.

After the workshop, between December 2014 and July 2015, participants applied their learning, sharing the TRP with colleagues, reviewing curricula and training methods, and identifying ways to strengthen family planning training at individual institutions. Some of the participants attempted to use the TRP as is and faced challenges with reducing the time and aligning the content with what is expected in preservice education. As a result, it was recommended to hold workshops in specific countries to align the TRP with their preservice education standards and to develop lesson plans.

Participants from Lesotho, South Africa, Tanzania, Uganda, and Zimbabwe asked that we support them to implement the TRP to strengthen family planning training in their countries. The participants also suggested that in any future trainings, we upload the TRP content to flash drives so that participants can access the modules even when there is no Internet.

### Analyzing Content of Preservice Education in ECSA Countries

After the workshop, we developed a questionnaire based on WHO's *Core Competencies in Adolescent Health and Development for Primary Care Providers*,^8^ and asked nurses and midwives from ECSA countries to complete it between February and April 2015. The questionnaire assessed treatment of family planning, youth sexual and reproductive health, and gender in curricula; the capacity of schools to teach family planning; teaching methods used; time allocated for both classroom instruction and practicum; and preparation of educators to teach family planning. A survey of nurses and midwives from ECSA countries revealed that countries dedicated insufficient time to comprehensive family planning training and that training focuses on provision of short-acting methods. Moreover, training was conducted mostly through lectures, rather than practicums, which is widely considered to be a less effective training modality. Based on this information, and with guidance from ECSACON, we chose to support the adaptation of the TRP for preservice education with nursing and midwifery preservice educators from schools in Tanzania and Uganda. Tanzania was in the process of reviewing preservice curricula and Uganda had plans to update preservice curricula—which were long past due for review.

A survey of nurses and midwives from ECSA countries revealed that countries dedicated insufficient time to comprehensive family planning training and that training focuses on provision of short-acting methods.

### Assessing Tanzanian and Ugandan Preservice Education

Once we selected Tanzania and Uganda, in May 2015, we conducted a more detailed review of preservice family planning education in these 2 countries. We held discussions with ECSA and representatives of nursing and midwifery councils and preservice education program managers in Tanzania and Uganda, and we conducted a desk review of preservice curricula and resources. The desk review consisted of relevant websites including those of Johns Hopkins University's Knowledge for Health Project, Human Resources for Health's Global Research Center, the Global Health Workforce Alliance, implementing partners known to have supported preservice education programs, and ECSA and ECSACON. Following the online search, country-specific documents were requested from Tanzania and Uganda, such as scopes of practice, curricula for in-service and preservice education, and family planning and reproductive health policies and guidelines.

Across both countries, nursing and midwifery councils accredit and regulate nursing and midwifery schools and determine curricula content. Content must then be approved by committees, which are typically composed of policy makers, educators, regulatory councils, content experts, and professional associations. Curricula must be reviewed and updated every 5 years, or as needed, based on changes in policies and new technology. Approved curricula are disseminated to the schools. Individual educators then design each training session using a standard template provided by the preservice training units of the Ministry of Health or Ministry of Education, leading to lack of standardization of the content taught, time allocated to teach family planning, and training methodology used. Both countries allocate 40 hours to the entire reproductive health module, which includes family planning, sexually transmitted infections, HIV/AIDS, adolescent and youth sexual and reproductive health, and postabortion care.

While training standards in Tanzania and Uganda specify that curricula have to be competency-based, educators in both countries tend to have limited experience in use of such training methods. However, both countries cited that educators have limited experience in use of competency-based training methods and tend to depend instead on lectures. The Tanzanian preservice family planning curriculum was updated in 2015, but Uganda's curriculum had not been revised for many years, some parts for more than a decade.

While training standards in Tanzania and Uganda specify that curricula have to be competency-based, educators in both countries tend to have limited experience in use of such training methods.

### Workshops

In 2015, we organized 2 workshops in each country. During these initial workshops, we disseminated the TRP, oriented participants on its structure, and distributed *WHO's 2015 Medical Eligibility Criteria (MEC)*^9^ *and the USAID High Impact Practices*.^10^

The first planning workshop in each country was 3 days long and attended by key stakeholders, including preservice education policy makers, family planning program managers, educators from nursing and midwifery training institutions, and representatives from professional associations and curricula-development committees. The aim of this initial workshop was to disseminate the TRP, foster country ownership, and identify gaps in preservice family planning education curricula that could be addressed through application of the TRP. Participants arrived at recommendations for improvement of preservice family planning education based on the country's policies and standards, including family planning knowledge, attitudes, and skills to be developed during preservice education and job expectations post-graduation. At the initial workshop, participants then developed the design and schedule for the subsequent 5-day adaptation workshop; for example, they decided which TRP modules should be used to demonstrate adaptation of the TRP. The demonstration modules were selected based on what content the educators felt they and their students needed to know more about, and what content needed an update within the curricula. Participants also developed the schedule and methodology for the adaptation process.

The second 5-day workshop in each country focused on adapting the selected TRP modules. The workshop included a broader set of participants to adapt the TRP: educators from public- and accredited private-sector schools for nursing and midwifery, regulatory councils, professional associations, in-service family planning trainers, and service providers from practicum training sites. Those who attended the 3-day planning workshop served as co-facilitators during the 5-day workshop. We documented our lessons learned for other program implementers to use when adapting the TRP to local contexts.

In both countries, we established an online community of practice to allow participants to interact with one another and support continued learning. However, we recognized that the limited Internet connectivity in Uganda would somewhat hamper these participants' ability to engage.

#### Tanzania

We held the Tanzanian 3-day planning workshop in July 2015. Participants decided on the following objectives for the 5-day adaptation workshop: (1) adapt the TRP to validate the newly updated family planning content; (2) demonstrate how to adapt the TRP using the counseling module as an example; (3) update the knowledge and skills of participants on family planning; (4) conduct contraceptive technology update; (5) demonstrate use of competency-based training methods; and (6) develop session plans for the updated curricula family planning module.

The Ministry of Health and Social Welfare led the 5-day workshop, and 33 participants attended including educators from the nursing and midwifery training institutions, universities, and local ECSACON country representatives. We showed participants how to access and use the TRP online and on flash drives. In-service family planning master trainers from the Ministry of Health presented sessions on the WHO's MEC and the TRP's counseling module, demonstrating use of competency-based training methods. Participants then adapted the existing curricula according to the TRP content. Table 1 shows the changes to the curriculum in Tanzania after the workshop.

**TABLE 1.** Changes to the Tanzanian Curriculum Before and After the TRP Workshop

Before Curriculum Review (2009)	FP Curriculum Content and Learning Outcomes Revised After the TRP Workshop (July 2015)
**Learning Outcomes**	**Learning Outcomes**
Provide FP services in the community	Provide FP services according to guidelines and protocols
**Content Outline**	**Content Outline**
Define FP	Define FP
Identify advantages of FP	Identify myths and misconceptions related to FP methods
Explain various methods of FP	Explain advantages of FP
Counsel clients on FP methods	Describe short- and long-acting reversible contraceptive methods
	Explain elements of FP service delivery
	Take obstetric and gynecological history
	Perform physical examination
	Counsel the client on informed choice
	Screen client for medical eligibility for contraceptive choice
	Initiate the chosen contraceptive method (oral contraceptive, injectable, implant, intrauterine devices, and natural and barrier methods)
	Plan for a follow-up visit
	Refer for permanent methods (vasectomy, tubal ligation) when appropriate

Abbreviations: FP, family planning; TRP, Training Resource Package for Family Planning.

We demonstrated how the trainers could adapt the TRP to inform their own lesson planning. To allow participants to practice, we divided them into groups of 3 and randomly assigned them a topic (e.g., balanced family planning counseling). Participants in the adaptation workshop used the TRP to develop lesson plans in accordance with national policies and guidelines in the standard templates. Once developed, participants presented their lessons to others and received feedback. Some adopted the session training materials and PowerPoint presentations directly from the TRP. Others removed slides that they felt were too advanced for their students. Still others made small modifications to the TRP based on their knowledge of students' needs and what is already taught through preservice education (e.g., communication skills, anatomy and physiology of the male and female reproductive organs). All participants used the TRP to create comprehensive lesson plans. Participants reported that they would have liked to have had more time to develop lessons plans and practice session delivery for the whole family planning module.

Participants in the adaptation workshop used the TRP to develop lesson plans in accordance with national policies and guidelines in the standard templates.

In Tanzania, all participants had a laptop, so while we provided flash drives with the TRP modules included, participants primarily accessed the TRP online. Participants expressed satisfaction with the TRP itself, especially its ease of use and the many resources included with it. Overall, they preferred didactic teaching methods, rather than practicum methods. They suggested that in future sessions, facilitators might spend more time demonstrating role-play and other competency-based methods before they asked participants to do so. Lastly, they found adaptation a challenge, reporting difficulty reducing the exhaustive TRP content to fit within the allotted 20 hours of time for family planning training.

Since the workshops, the National Pre-Service Education Coordinating Unit of the Ministry of Health and the Nurses Council aligned competencies in the diploma nursing and midwifery curricula with global standards, using the TRP as a benchmark. The National Council for Technical Education (NACTE) approved the updated curricula. The Coordinating Unit then organized workshops to orient 139 educators representing training institutions for nurses and midwives in Tanzania.

#### Uganda

In December 2015, we hosted the 3-day planning workshop and subsequent 5-day adaptation workshop in Uganda. Based on feedback from participants, we then added a third, 2-week workshop where participants from the 5-day workshop reconvened to develop lesson plans that used Uganda's standardized template for preservice education. During the workshops, these lesson plans, along with supporting materials, were compiled into a *Trainers' Reference Guide for Family Planning Pre-service Education for Nurses and Midwives*, further described below.

During the 3-day workshop, participants decided on the following objectives for the 5-day workshop: (1) review the TRP to ensure its alignment with national policy; (2) update the preservice modules on contraceptive technology and competency-based training methods; and (3) standardize lesson planning.

Thirty-two representatives of nursing and midwifery schools, the Uganda Nursing and Midwives Council, the Uganda Nursing and Midwifery Examination Board, and ECSA attended the 5-day workshop in Uganda. The Nursing and Midwifery Examination Board, which is under the Ministry of Education, was included instead of the Ministry of Health (as in Tanzania), because in Uganda the Ministry of Education has jurisdiction over preservice education. Participants spent the first few days of the Ugandan workshops reviewing the nursing and midwifery curricula to determine content gaps and needs. As the curricula had not been reviewed since 2003, participants viewed the TRP workshops as a useful opportunity to undertake a thorough curricula review of the entire reproductive health course unit's family planning objectives and content. We disseminated the TRP online and on flash drives. Based on the recommendations from the 3-day workshop to use modules with content that would be new to most of the participants, we demonstrated use of the TRP using modules on emergency contraceptive pills and the Standard Days Method. The use of role-plays and demonstration enhanced participants' learning and reinforced competency-based training methods.

In small groups, participants cross-checked existing preservice curricula against the reproductive health unit family planning standards, in-service training curricula, and TRP content. During this examination, they revised learning objectives and updated knowledge, skills, and attitudes to be developed during preservice education. They then developed the content outline, resulting in a much more comprehensive curriculum (Table 2).

**TABLE 2.** Changes to the Ugandan Curriculum Before and After the TRP Workshop

Before Curricula Review (2005–2008)	Revised Objectives, Competencies, and Content After the TRP Workshop (December 2015)
**NURSING**	**NURSING AND MIDWIFERY**
**FP Objectives**	**Objectives**
Describe all FP methods	Identify clients for FP/RH services
**Competencies**	Communicate and promote FP/RH effectively to different population groups
Provide all FP methods	Counsel clients for voluntary informed choice
**Content Outline**	Provide clients with oral pills, progestin-only injectables, ECPs, implants, IUDs, SDM, Cervical Mucus Method, and barrier methods according to national FP/RH guidelines
Define FP	Integrate FP with other services including MNCH, STIs, and HIV/AIDS
History of FP	Identify clients with FP/RH complications
Benefits and disadvantages of FP	Manage clients with FP/RH complications
Management of FP services	Manage FP clients with STIs and HIV/AIDS
**MIDWIFERY**	Refer clients to other FP/RH services appropriately
**FP Objectives**	Document, manage, and utilize data related to FP/RH
Assess clients for different FP methods	**Content Outline**
Explain FP services	Define FP
**Competencies**	Benefits of FP
Counsel clients on FP	Rights-based FP/RH service delivery
**Content Outline**	Counseling for FP and voluntary informed choice
History of FP	Cultural beliefs and practices related to FP
Benefits and disadvantages of FP	Methods of FP/contraceptive technology (oral pills, progestin-only injectables, ECPs, implants, IUDs, SDM, Cervical Mucus Method, condoms – male and female, other barrier metohds)
Management of FP services	Medical Eligibility Criteria for contraceptive methods
Monitoring and evaluation of FP services	Provision of FP methods
	Elements of successful FP monitoring, FP/RH service delivery
	Provision of FP for special groups (adolescents, postpartum clients, postabortion care, HIV/AIDS, ending mother-to-child transmission of HIV, men)
	Myths and misconceptions of FP

Abbreviations: ECPs, emergency contraceptive pills; FP, family planning; IUDs, intrauterine devices; MNCH, maternal, newborn, and child health; RH, reproductive health; SDM, Standard Days Method; STIs, sexually transmitted infections; TRP, Training Resource Package for Family Planning.

Once the objectives of the family planning curricula and content on knowledge, skills, and attitudes were established, the small groups allocated time to each objective, developing lesson plans and incorporating competency-based training methods. In Uganda, few participants had computers, so hard copies of the TRP modules were printed and distributed to participants to use in their small groups.

Participants provided valuable feedback. Some workshop participants found it challenging to adapt the extensive TRP content into the limited time allotted for preservice education. They suggested that we develop more explicit guidance on how to use the TRP to develop preservice education lesson plans. Disseminating the TRP to all schools in the country would be helpful, participants said, in standardizing training content.

Some workshop participants found it challenging to adapt the extensive TRP content into the limited time allotted for preservice education.

In July 2016, a follow-up, 2-week workshop was organized in Uganda to adapt the TRP to develop lesson plans. During this workshop, we worked with participants to adapt the TRP to develop the *Trainers' Reference Guide for Family Planning Pre-service Education for Nurses and Midwives*. The Reference Guide includes a series of predesigned lesson plans that used Uganda's standardized template for preservice education; PowerPoint presentations; handouts; and knowledge evaluation questions that could be adapted to develop pre-post knowledge tests, quizzes, and skills assessment checklists. The Reference Guide includes guidelines for practicum training, development of practicum training sites, and preparation for practicum training. The Trainers Reference Guide is aligned with the TRP, and cognizant of the time allocated to family planning classroom teaching. Based on recommendations from the 2015 workshops, participants were given more time to practice delivering their sessions. They “pretested” lesson plans and used feedback from facilitators and peers to inform revisions. They added some lesson plans that are not part of the TRP, such as the family planning/reproductive health policies, guidelines, and strategies, and sessions on “family planning concepts” and permanent methods for enhanced knowledge on country context and links between family planning and maternal, newborn, and child health.

Since developing the lesson plans, the National Curriculum Development Committee and educators who had attended the TRP workshops reviewed the certificate and diploma nursing and midwifery preservice education curricula, enabling the TRP to be a reference document. The lesson plans and Reference Guide have been disseminated to training institutions. The training institutions conducted workshops with other educators who had not attended the TRP workshops facilitating use of the lesson plans. The educators found the lessons plans to have sufficient content for preservice education.

## RESULTS AND OBSERVATIONS

Adaptation of the TRP in Tanzania and Uganda resulted in substantive changes to the curricula of the reproductive health course unit that will support nurses and midwives to provide quality, rights-based family planning (Table 1 and Table 2). In both countries, we asked participants to take pre-post knowledge tests to assess knowledge gained throughout the course of the workshop. The pre-post tests showed significant gains in family planning knowledge: the average pre-test score was between 30% and 40%, and the average post-test score increased to more than 80%.

Adaptation of the TRP in Tanzania and Uganda resulted in substantive changes to the curricula of the reproductive health course unit that will support nurses and midwives to provide quality, rights-based family planning.

### Sustainability

Six months post-workshop, we checked in with workshop participants to assess whether the TRP was still in use. In Tanzania, educators who had attended the workshop were using the TRP and had shared the TRP and adapted lesson plans with their colleagues. The Ministry of Health had used the TRP as a reference to develop curricula for the country's newly formed community health worker cadre. Workshop participants were using the TRP resources and tools (most notably, the PowerPoint presentations) in their classrooms; however, they reiterated the challenge of reducing content from the TRP to fit within the allotted amount of time for preservice family planning training. Because the Uganda lesson plans were developed within the time allotted to the reproductive health course unit, educators in Uganda did not experience the same challenge as those in Tanzania. However, they did express challenges in using competency-based training due to large class sizes and lack of adequate training resources, such as anatomic models. In Uganda, participants reported using the TRP and again affirmed the value of the Trainers' Reference Guide, which helped to standardize learning and contained lesson plans. Although educators who attended the workshops shared the TRP and adapted lessons with their colleagues, we do not know how the resources are being used by schools that were not part of the workshops.

Our workshops revealed some areas for improvement within the TRP. Any future revisions may consider adding content to address the following topics in greater depth:

- Basic foundational concepts for family planning service delivery, such as the meaning of healthy timing and spacing of pregnancy and rights-based family planning
- Strategies for deconstructing myths and misconceptions about family planning
- Recordkeeping and health data management including data use to improve family planning service delivery
- Gender and adolescent and youth sexual and reproductive health

### Lessons Learned

Through the process of adapting the TRP, we learned several lessons that may be useful to other implementers as they implement the TRP in their own countries.

- The process of reviewing the TRP with key stakeholders is a learning opportunity. Implementers of preservice education programs should organize content review workshops with educators and representatives of relevant ministries, allowing sufficient time for participants to identify and discuss in detail differences in their existing curricula and the TRP.
- Similarly, productive relationships with ministries of health and education, regulatory councils, and professional associations in each country proved invaluable for rolling out the TRP adaptations. Inclusion of nursing and midwifery educators in the adaptation process helped to create an established pool of trainers who could then cascade the TRP to other educators.
- Adaptation of the TRP for preservice education should be context specific. Adaptation is a complex process and not one process will fit the needs of all countries. It is therefore important for stakeholders involved in the adaptation process to have a thorough understanding of country context.
- Our experience reinforced our belief that high-quality preservice education is a crucially important element of building nurses' and midwives' foundational skills. But educators are only as good as the tools and resources available to them. It is critically important that educators' skills in contraceptive technology and competency-based training methods be routinely updated and they are equipped with technically accurate textbooks.

Inclusion of nursing and midwifery educators in the adaptation process helped to create an established pool of trainers who could then cascade the TRP to other educators.

## CONCLUSIONS

Strengthening preservice family planning education for nurses and midwives can improve health outcomes for women, newborns, infants, and children. Quality preservice family planning education for nurses and midwives is therefore a “best buy” for countries seeking to reduce maternal, newborn, and child mortalities and morbidities. A global analysis conducted by UNFPA in 2014 concluded that midwives, when educated to international standards, have the competencies to deliver 87% of the 46 essential reproductive, maternal, and newborn health services needed by women and newborns.^11^ The TRP, as an evidence-based tool, can be applied and adapted globally to improve the quality of family planning service delivery and respond to the need to improve the global health workforce.

### Next Steps

Based on our experience in Tanzania and Uganda, we developed 2 useful tools for those interested in replicating the process described in this article. The first is a *How-To Guide* for adaptation of the TRP to improve preservice family planning education (see https://www.e2aproject.org/publication/guide-adaptation-training-resource-package-family-planning-improve-pre-service-education/). The *How-To Guide* contains detailed steps elaborating the adaptation process, including examples of adapted training modules. The second is a *package of modules*—one for preservice family planning education and one for preservice education on adolescent and youth sexual and reproductive health and gender (see https://www.e2aproject.org/publication/training-resource-package-pre-service-education-family-planning-adolescent-youth-sexual-reproductive-health/). Adolescent and youth sexual and reproductive health is taught in its own standalone unit in both countries, but the broader curricula do not discuss age biases in family planning service provision. The modules contain lesson plans and supporting materials that can be used by educators to teach essential family planning, adolescent and youth sexual and reproductive health, and gender competencies to nurses and midwives during the time allotted for preservice family planning education.

## Notes

**Peer Reviewed**

**Funding**: The Evidence to Action (E2A) Project, under Award Number AID-OAA-A-11-00024, gratefully acknowledges the generous support of the U.S. Agency for International Development for the development of the Training Resource Package for Family Planning (TRP), dissemination of the TRP, and adaptation of the TRP in Tanzania and Uganda.

**First Published Online:** August 30, 2018
